# Off pump surgical epicardial closure of left anterior descending to pulmonary artery fistula

**DOI:** 10.1186/s13019-020-01329-2

**Published:** 2020-10-08

**Authors:** Gustavo L. Knop, Ernest Madu, Edwin Tulloch Reid, Ahmed Soliman

**Affiliations:** 1 Cardiothoracic Surgeon, FRCS, Heart Institute of the Caribbean, Kingston, Jamaica; 2 FACC, FESC, FRCP, Heart Institute of the Caribbean, Kingston, Jamaica; 3 Interventional Cardiologist, FACC, Heart Institute of the Caribbean, Kingston, Jamaica; 4 Interventional Cardiologist, FESC, Heart Institute of the Caribbean, Kingston, Jamaica

**Keywords:** Coronary artery to pulmonary artery fistulas, Coronary artery anomalies, Coronary artery fistulas causing myocardial ischemia, Surgical closure of coronary artery fistulas

## Abstract

**Background:**

Coronary artery fistulae (CAF) are rare anomalies. Left anterior descending(LAD) to Pulmonary artery (PA) CAF, represent a minority of cases. Large fistulas, create a significant shunt and a “steal phenomenon”, and can lead to myocardial ischemia and heart failure (HF) if left untreated.

**Case presentation:**

We present a 57 years old female with a large LAD to PA fistulae. Given the rare occurrence and the predominance of low shunt of LAD to PA CAF, this case is functionally exceptional in this fistulae variant, causing a significant shunt which resulted in daily cardiac ischemic chest pain. Diagnosis work up included a nuclear stress test, Coronary Angiography and 3-D Coronary Computed Tomography Angiogram (CCTA). Traditionally, surgery has been the main therapy for symptomatic CAF, but transcatheter closure has emerged as a less invasive strategy and is a valuable alternative or even preferable if no associated cardiac conditions are present, provided the anatomical characteristics of the fistulae are appropriate. The surgical approach includes off pump epicardial interruption of the fistula or closure through a cardiac chamber (trans-cameral) or transpulmonary, or epicardial closure using Cardiopulmonary bypass. Caution must be taken in cases of CAF with Coronary Artery (CA) aneurysm in dominant CA, or drainage into the Coronary Sinus, as the possibility of ischemic complications are higher. Due to anatomical considerations and tortuosity of the fistulae, our patient was considered not amenable for percutaneous closure and surgery was opted. Epicardial closure of the fistula was performed on a beating heart, off pump. Outcome was favorable with complete resolution of ischemic symptoms.

**Conclusion:**

Symptomatic, high shunt CAF must be interrupted. The presence of daily ischemic symptoms in our case report patient, is worth to be remarked. Alternatives for fistulae closure are transcatheter or surgery, depending on anatomic variables and the presence of associated cardiac conditions. Surgical epicardial closure of LAD to PA fistulae variant can be done with very low mortality and morbidity, but other variants with coronary aneurysm, drainage in the coronary sinus or other concomitant cardiac defects, may result in ischemic complications and higher perioperative mortality and worse long- term outcome.

## Background

Coronary artery fistulae (CAF) are rare anomalies that consist of congenital or acquired vascular communications between the coronary vessels and the cardiac chambers or other vascular structures, such as vena cava, the pulmonary artery, or pulmonary veins. Their incidence in the overall population is reported about 0.002% [1].

Large fistulas, create a significant shunt, as the blood follows the lower resistance pathways rather than traversing the smaller arterioles of the myocardium. This flow pattern can lead to heart failure if left untreated.

There can be considerable variation in the course of a CAF.

Most of the patients remain asymptomatic, but symptoms and complications may develop with increasing age, as the caliber of the fistula tend to enlarge. Patients may present with myocardial ischemia, myocardial infarction (MI), congestive heart failure, or sudden death [2].

CAF are often diagnosed by coronary angiogram.

However, with the advent of new technologies such as CCTA, the course and communications of these fistulae can be delineated non-invasively and with greater accuracy.

Management options of patients with coronary artery fistulas depends on the individual case.

Guidelines mandate a multidisciplinary team approach to discuss the type of therapy.

## Case presentation

We present a case of CAF between the proximal LAD and the PA trunk [3], causing a “steal phenomenon” to distal LAD territory. Given the rare occurrence of this fistulae type, the clinical presentation is exceptional in this variant, causing a significant shunt from the LAD territory, which resulted in daily cardiac ischemic chest pain.

**Fig. 1:**
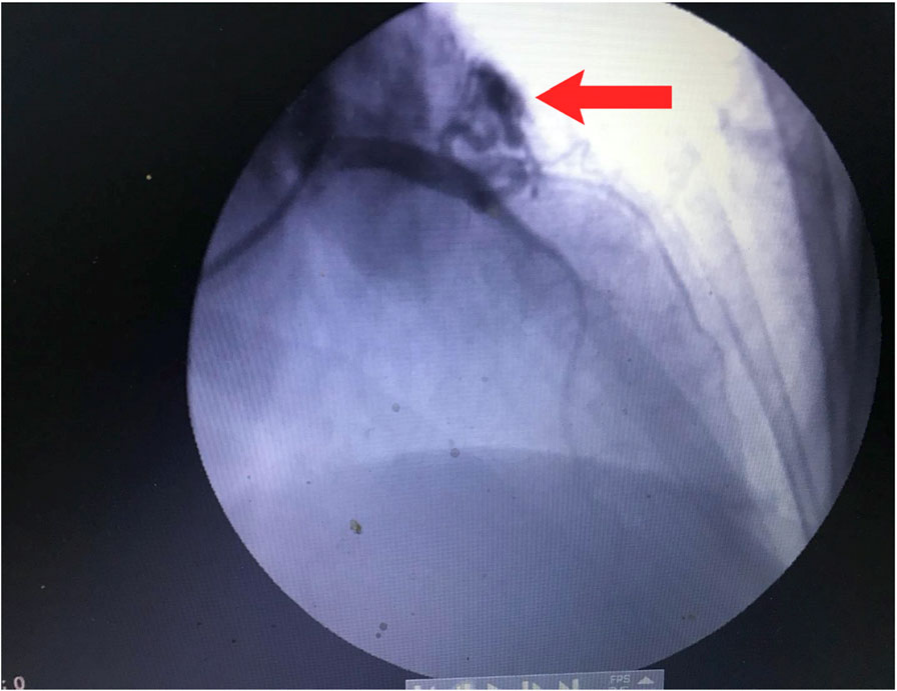
Coronary angiogram: LAD – PA Fistula (red arrow)

A 57 years old female, with Type 2 DM, presented to consultation complaining of typical anginal chest pain for 2 years. Anginal episodes were related to physical activity and occasionally at rest, had variable duration and were associated with dyspnea and dizziness. During the last 6 months, symptoms progressed to become daily despite optimal medical treatment. Physical examination was unremarkable. Electrocardiogram showed normal sinus rhythm and no ST changes, Trans-thoracic echocardiogram, on Doppler analysis, revealed a small jet flow in the main pulmonary trunk without definite identification of its source. We decided to proceed to a nuclear stress test to confirm ischemic heart disease. The test revealed an antero-apical wall defect, along with normal left systolic function. Coronary Angiogram demonstrated the tortuous and dilated LAD to PA CAF, and of notice, a significant decrease in the caliber of the LAD distal to fistula take off, as an indirect sign of an ongoing “steal phenomenon”. LAD proximal to fistulae take off, was dilated. According to Sakakibara fistulae classification, this patient had a Type A fistulae (Fig. 1).

**Fig. 2:**
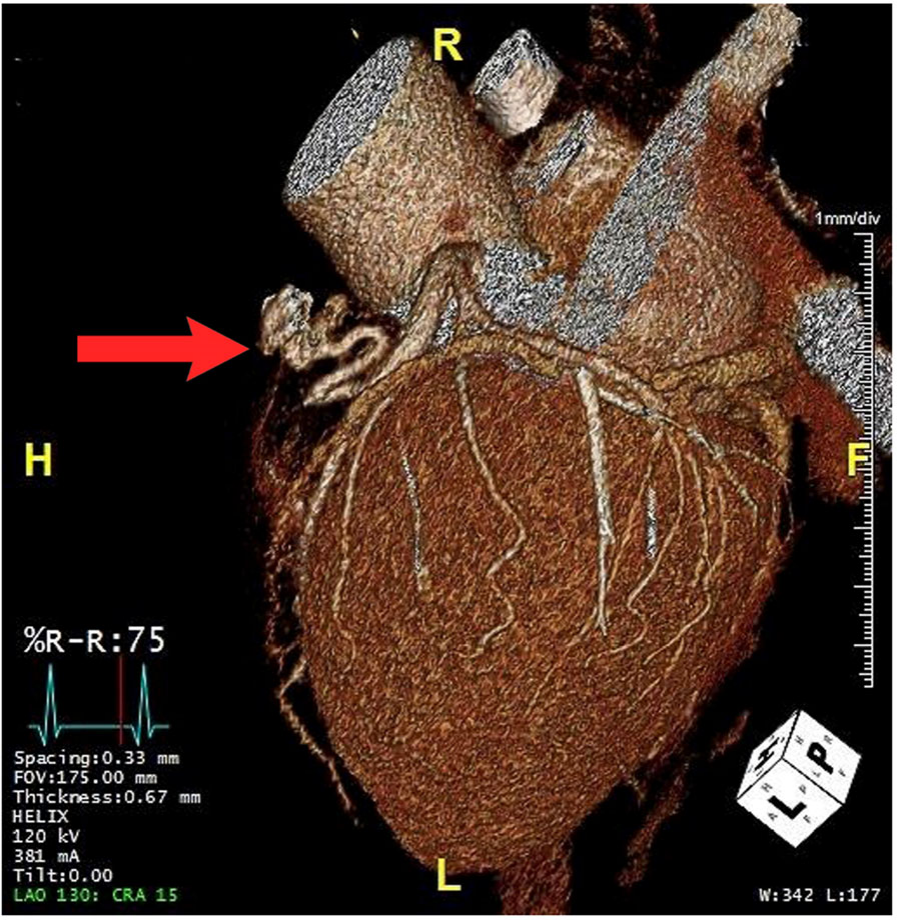
3-D CCTA imaging reconstruction of the LAD and PA fistulae and their insertion point to the PA (red arrow)

These types of fistulae are typically complex and large, and may have a high flow [4]. 3-D CCTA was performed, which confirmed the isolated aberrant branch (fistula), taking off from the LAD at 0.4 cm distal to first diagonal branch, which communicated with the left anterior surface of the pulmonary trunk. The fistula was very tortuous and measured 9.96 cm in length (Fig. 2).

Because of the anatomical location and characteristics, the tortuosity of the trajectory, the caliber of the fistula, and the severity of the symptoms, surgical closure was decided [4].

The operation was performed through a standard median sternotomy, on a beating heart, OFF-PUMP.

The fistula was clearly identified on the surface of the right ventricle reaching the proximal portion of the main pulmonary trunk above the pulmonary valve. The aspect of the fistula vessel wall, although of good caliber, looked very friable and easy to be ruptured on a beating heart. So, dissection and surgical clip closure was discarded. Instead, the fistula was closed using several stitches of 5/0 polypropylene with pledgets. Additional 5/0 stiches were placed on small branches running on surface of the main PA, suspicious of being branches or collaterals from the main fistula trunk. One fine branch running over RVOT was left intact, believed to be a branch of the RCA supplying the RVOT, visualized in the Coronary angiography.

LAD was not identified at the level of the sutures, due to its intramuscular location, but close observation of the monitor for the presence of transient ECG changes, and the anterior/septal wall motion during the procedure, precluded any accidental inclusion of LAD in the stitches. Postoperative outcome was uneventful, with no perioperative MI or other complications. The patient was discharged on the fourth post-operative day. She is currently pain free and with no dyspnea after 5 months of follow up.

https://www.dropbox.com/s/wate24fqx00e50k/HIC%20Surgery%20Summary.mp4?dl=0.

**Video 1.** CT Angiogram and Surgical Procedure.

## Discussion and conclusions

The severity of the shunt, presence of other cardiac conditions, and the location of the fistula, would be the determinants of the clinical presentation. In a review of 363 cases of CAF, 42% arise from the Left Coronary and only 17% drain in the PA. From those (17%), according to Said et all review [5], LAD to PA variable, represented only 26%.

There has been no definitive consensus for the management of CAF in the American Heart Association and American College of Cardiology Guidelines 2018 for adults with congenital heart disease, although a conservative approach predominated. Review of published reports established that closure is indicated for symptoms of HF or Myocardial Ischemia and for those asymptomatic patients with significant shunt or large fistulas that create a risk for future complications, such as infective endocarditis, pulmonary hypertension or HF [1].

Our case report patient had daily episodes of chest pain associated with dyspnea and dizziness, which progressed in the last 6 months before surgery. Therefore, in our view, closure of the CAF was clearly indicated.

CAF can be closed by surgical methods or transcatheter techniques [6]. There is little data to compare them, as the volume of cases is insufficient for a randomized trial, and there is no preference for either route.

To proceed with percutaneous closure, the body of the fistula must present a suitable landing zone for the selected device (coils, vascular plugs, or covered stents). The landing zone must be some distance away from the fistulae ostium to avoid native coronary artery in situ thrombosis and/or distal embolization with the subsequent occurrence of periprocedural or late MI. Tortuosity of the fistula is an additional characteristic to take into account for decision making, compromising the success of percutaneous approach [4].

In the present case, the CAF was very tortuous, and the curves precluded a safe landing zone distant from the fistulae take off from the LAD. In multidisciplinary meeting discussion, our expert interventional cardiologists considered this type of CAF not amenable for a safe transcatheter closure.

Surgical closure of CAF can be performed with or without Cardiopulmonary Bypass, isolated or associated with other surgical procedures if they were indicated.

Multiple surgical techniques were described for CAF closure, such as epicardial ligation or division, or closure through a cardiac chamber (transcameral) or transpulmonary approach.

We opted for off pump epicardial interruption of CAF using several 5/0 polypropylene stiches with pledgets due to the fragility of fistulae wall. Temporary clamping of the fistulae to assess myocardial ischemia was considered not advisable, due to fragility of the CAF. Complications of Surgical procedures described in the literature were related to perioperative and late MI, reaching 11% in one study [5], which resulted in subsequent reduced late survival with age-matched groups. Despite that, more than 90% of the late mortality cases in the study was from non- cardiac causes. These results may reflect a selection bias, because fistulae referred for surgical closure are usually not amenable to percutaneous closure or are associated with comorbid cardiac conditions requiring surgery. Moreover, adverse outcomes were more frequently associated with large and significant CAF and aneurysmal Coronary Arteries (CA). Drainage of CAF into the Coronary Sinus was significantly linked with a major complication occurrence. None of these alternatives were present in our case report, neither transient ST changes or wall motion abnormalities were noted during or after CAF closure.

## Conclusions

Symptomatic, high shunt CAF must be interrupted to avoid ischemia, pulmonary hypertension or cardiac failure. The presence of daily ischemic symptoms correlated to a steal phenomenon in the LAD territory, caused by a significant shunt through the CAF.

The decision of which modality to use, even percutaneous transcatheter or surgery, should be based on the anatomical location of coronary artery fistulas and concomitant cardiac conditions that need treatment.

Transcatheter closure approaches have emerged as a less invasive strategy and are considered at present, a valuable alternative and even preferable to surgical correction in favorable locations and trajectory of fistulas, in which a secure landing zone can be established to refrain from complications like CA thrombosis or distal embolization. The surgical approach of CAF may be done off pump or with the use of Cardiopulmonary bypass, associated if needed with other cardiac surgical procedures. Caution must be taken in cases of CAF with CA aneurysm in dominant CA, or drainage into the Coronary Sinus, as the possibility of ischemic complications are higher. LAD to PA CAF variant can be surgically closed off pump, with very low morbidity and mortality. This technique precludes any chance of recanalization if properly accomplished.

## Data Availability

“Not applicable”.

## Abbreviations

CAF: Coronary artery fistulae
LAD: Left anterior descending
PA: Pulmonary artery
HF: Heart failure
CA: Coronary artery
CCTA: Coronary Computed Tomography Angiogram
MI: Myocardial Infarction
